# Enlarged Prostate, Vesical Catarrh and Consequent Renal Disease

**Published:** 1872-11

**Authors:** J. A. Mowris

**Affiliations:** Syracuse, N. Y.


BUFFALO

### Medical and Surgical Journal.

VOL XII. NOVEMBER, 1872. No. 4

### Original Communications.

——:o:——

ART. I.—*Enlarged Prostate, Vesical Catarrh and Consequent Renal Disease.* By J. A. Mowris, M. D., Syracuse, N. Y.

It is unfortunate, that the true significance of many a medical case and its possible value to science, cannot be known till made plain by post mortem revelation, when the history of the case in its minutia and natural sequence is perhaps unavailable.

The following case of disease, involving the health and life of one of our most exemplary and influential citizens, possesses a medical interest which amply entitles it to a place in medical literature.

E. B. W., Banker, aged 56, of good habits and excellent general health. The earliest symptoms of derangement was irritation in the neck of the bladder. The urgency of this symptom gradually increased until, in the course of a year, his sufferings from this cause became very severe. There was frequent and painfully urgent desire to evacuate the bladder, with utter inability to do so. This vesical tenesmus was attended with involuntary expulsive efforts of such force as to induce a motion of the bowels. The frequent recurrence of this distressing symptom and consequent exhaustion, at length, disqualified him for the pursuit of his business, and he was forced to relinquish it. At this stage he fell into a state of despondency, characterized by aversion to society. So intense was this feeling that at times the presence of his most intimate friends was intolerable. He preferred and sought the solitude of his own chamber.

Quite early, his physician was called. Examination disclosed enlarged prostate gland. A resort to the catheter was had, affording great, but temporary, relief. It being evident to the physician that the patient could not empty the bladder without artificial aid, he was instructed, and by that means thereafter relieved himself.

The urgent desire for voiding the urine recurred as often as once in two hours, night and day, and at times much oftener, during the remaining four years of his life, he always responding to the call by the use of the catheter.

The introduction of a sound failed to elicit indications of stone. At this time, something more than a year after his attack, he complied with the suggestion of his physician and other friends, and sought the advice of eminent physicians in New York City. He obtained of them very little satisfaction and no encouragement. Dr. Van Buren, of that city, diagnosed enlarged prostate gland and consequent vesical catarrh, recommended frequent flushing or washing of the internal surface of the bladder with water injections, and expressed the opinion that the derangement would result in fatal renal disease.

His New York physicians concurred in recommending a tour in Europe, in the hope that diversion of mind and necessary exercise would reassure his failing nervous system. Accordingly, on the 25th day of April, 1868, about two years after his attack, accompanied by his wife and son, he set out, taking quite an extensive trans-Atlantic tour. He consulted Simms, of Paris, Thompson, of Edinburgh, and others, eliciting only a repetition of the first diagnosis, but obtained no encouragement.

Though this journey of six months' duration effected no abatement of his local disease, his general health was evidently improved thereby. At times during the tour he was unusually cheerful and quite himself. If his excursion was not remedial to his primary disease, it certainly rendered his remaining days much more tolerable.

His most severe discomfort occurred from four to six o'clock A.

M. These daily paroxysms not unfrequently deranged his plans for resuming his journey. There was also a period of increased distress as often as every tenth day. The punctuality with which these recurred imparted the feature of periodicity.

At intervals of three or four weeks, the discharge from the bladder was a fetid and offensive mixture of blood and pus. Some six months before his death he was seized with chills, which re-appeared at intervals till the final scene.

Anodynes had not been much resorted to till about ten months prior to his death, when chloral hydrate was employed with fine effect. A dose of grs. xv, or grs. xx. procured for him an hour or two refreshing sleep, and to this agent he never resorted in vain till within a day or two of his last hour, when its effect became uncertain and unsatisfactory.

For two or three months there was difficult defecation, due evidently to obstruction by the prostate gland.

Over the region of the left kidney, at length, there appeared a well marked prominence, painful and tender to the touch. It presented the appearance of abcess, as if pointing for external issue. This occurred about six weeks prior to the end, when the powers were rapidly failing. The prominence never matured sufficiently to justify incision.

For two or three months he had been perceptibly declining in strength, though he continued to walk into the yard and about the house, sitting at table and conducting family worship with much regularity; but, on the first day of the present year, he seemed exhausted and unable to rise. For a fortnight he slept only under the influence of choral, and was never again dressed. From this time forth, the evacuation of the bladder was repeated by himself in response to the urgent desire as often as once in fifteen minutes, though the quantity of water was scarcely more than a few drops.

Aphthous sore mouth was latterly super-added to his ills. Severe chills, at short intervals, and an agony which chloral could not control. He sank on the 3d day of February.

Albumen was never detected in the urine—no noticeable anasarca—no dyspepsia. There was wakefulness, but never drowsiness nor stupor.

POST MORTEM APPEARANCES

Twelve hours after death. Body well nourished, complexion sallow, with waxy appearance of the surface—rigor mortis normal. Natural texture of right kidney entirely absent; substitution of fatty formation. Radical incision disclosed large quantity of pus. Left kidney similarly degenerated, but little normal structure remaining; also filled with pus. Bladder contracted to a capacity of perhaps three ounces. Coats much hypertrophied. The bladder also contained pus, and three phosphatic calculi, averaging the size of small filberts. Prostate gland at least four times the normal size, and in a state of profuse suppuration. The pelvis and the renal region were filled with a substance analogous with the fatty degeneration into which the kidneys had been lost.

Many interesting scientific suggestions might be appended to so instructive a case, but their presentation is hardly necessary, as they must readily and variously occur to the average medical mind.

OCTOBER 16th, 1872.

